# Water-Resistant Surface Modification of Hydrophobic Polymers with Water-Soluble Surfactant Additives

**DOI:** 10.3390/polym13193407

**Published:** 2021-10-03

**Authors:** Colin P. Gibson, Matthew A. Litwinowicz, James P. Tellam, Rebecca J. L. Welbourn, Maximilian W. A. Skoda, Jan Claussen, Richard L. Thompson

**Affiliations:** 1 Department of Chemistry, Durham University, Durham DH1 3LE, UK; c.p.gibson@durham.ac.uk (C.P.G.); matthew.a.litwinowicz@durham.ac.uk (M.A.L.); 2 STFC ISIS Neutron and Muon Source, Rutherford Appleton Laboratories, Didcot OX11 0QX, UK; james.tellam@stfc.ac.uk (J.P.T.); becky.welbourn@stfc.ac.uk (R.J.L.W.); Maximilian.skoda@stfc.ac.uk (M.W.A.S.); 3 Procter & Gamble, German Innovation Center, Sulzbacher Str. 40, 65824 Schwalbach am Taunus, Germany; claussen.j@pg.com

**Keywords:** surface modification, hydrophobicity, blooming, segregation, surfactant, poly(isoprene)

## Abstract

Water-soluble nonionic surfactant, pentaethylene glycol monododecyl ether, C_12_E_5_, spontaneously blooms to the surface of spin-cast hydrophobic polyisoprenes, generating hydrophilic surfaces. This system provides a simple model for hydrophilic chemical modification of rubbery polymers that demonstrates surprisingly rich, complex, and unexpected behaviour. The vertical depth profiles were quantified using neutron reflectometry (NR) using a novel procedure to account for undulations in the film thickness. Surface properties were characterized using contact angle analysis and atomic force microscopy (AFM). Despite the low surface tension of the toluene solvent used in film preparation and the low surface energy of the polyisoprene (PI) matrix, NR depth profiles revealed clear evidence of surfactant segregation. This surface layer was typically thicker than a monolayer, but incomplete, yet was remarkably stable with respect to dissolution, even when exposed to hundreds of thousands of times the volume of water required to dissolve all the surfactant on the surface. Despite the apparent resistance to removal from the surface, water exposure does alter the subsequent wettability of the surface, with a hydrophilic-to-hydrophobic transition occurring after rinsing. Complementary AFM images of these C_12_E_5_/*cis*-PI films showed unexpected strand-like features on the surface of the film, which we attribute to a non-uniform lateral distribution of some of the surfactant. This surface structure becomes more evident after rinsing, and it appears that there are two distinct populations of surfactant on the PI film surface. We conclude that some of the bloomed surfactant exists as layers, which are relatively inert with respect to rinsing or surface modification, and some is laterally inhomogeneous. This latter population is primarily responsible for surface wetting behaviour but is not detected by specular NR.

## 1. Introduction

Polyisoprene, PI, is a key component of many industrial and consumer products; it is the main component of natural rubber, for which global annual production is around 10,000,000 tons [1,2]. However, for many applications, polymers need surface modification to satisfy requirements such as wettability, anti-fouling, or adhesion. There are a wide range of techniques to modify surfaces, including plasma induced grafting of acid groups [3], haloform groups [4] and fluorinated groups [5] as well as UV-cured surface treatments [6] with a range of surface modification groups such as fluorinated groups [7]. These techniques can be divided into physical and chemical techniques [2,8] and can help to impart desirable surface properties. 

Physical surface modification techniques involve the modification of the matrix polymer itself. These techniques include plasma [5] and ozone treatments [6], as well as flame and corona treatment methods. While these techniques can selectively modify specific areas of the surface, they introduce additional steps into the manufacturing process and frequently have high energy requirements. Furthermore, when used to generate hydrophilic surfaces, the benefits are often only temporary, with the polarised surfaces rearranging to minimise the surface energy [9]. This is likely to be particularly problematic for rubbery polymers such as PI, because of the high mobility of the polymer chains at ambient temperatures.

Chemical surface modification relies on the introduction of additive molecules to a polymer. Successful surface modification requires the delivery of these additives at the surface, which in turn requires some extent of segregation or demixing normal to the surface. The demixing of the two molecules is governed by the balance between the energetics of the interactions between the two components as well as the entropy of the formation of a segregated system [10,11,12]. Highly branched additives have shown promise in laboratory scale tests, where the partitioning of chain ends to the surface has been shown both experimentally [13,14] and computationally [15] to be entropically favoured through both Monte Carlo [11] and molecular dynamics approaches [16]. 

While it is relatively straightforward to generate hydrophobic surfaces, hydrophilic surfaces are more challenging to form because hydrophilic moieties usually have a high surface energy, which inhibits their surface segregation. Usually, small differences in surface energy along with incompatibility are most significant in directing and promoting surface segregation [17,18,19]. Previous methods to achieve this surface segregation have involved the use of amphiphilic additives, which combine low surface energy groups or low molecular weight to promote surface segregation, along with polar groups that could offer a hydrophilic surface property [19,20]. 

Surface modification of PI with amphiphiles has received relatively little attention, [21] and we are unaware of any which study this behaviour from a molecular scale perspective. There is, however, strong evidence of surface segregation of non-ionic surfactant in polypropylene, a more crystalline polymer than PI. These non-ionic surfactants have included stearyl alcohol ethoxylates [22], nonylphenol ethoxylates [23] and Irgasurf HL560 [24]. It has been shown that the structure of the additive molecule affects the migration of the additive: additives with a larger hydrophilic group (and thus greater HLB value) produce a larger surface layer [23]. However, the rate of surface migration becomes slower with increasing hydrophilic group size, possibly because this size hinders diffusion through the matrix to the exposed surface. We note that other contributing factors such as a greater tendency of larger molecules to form aggregates may also hinder the dynamics of surface segregation. 

The X-ray photoelectron spectroscopy results reported by Datla et al. [23] also showed surface segregation of non-ionic surfactants on polypropylene surfaces, and interestingly, the hydrophilicity imparted by the surfactants additive changed with ageing. For some surfactants, hydrophilicity increased with time, whereas for other surfactants this surface modification decreased with time. Unfortunately, it was not possible to resolve the composition profile either vertically or laterally, so it was not possible to relate changes in surface modification to the underlying structure of the adsorbed surfactant.

Here, we consider a model system of a hydrophobic polymer, *cis*-polyisoprene “*cis*-PI” and surfactant additive, pentaethylene glycol monododecyl ether “C_12_E_5_”. By using a simple single-component surfactant, it is possible to interpret nano-scale organisation and the blooming process at a molecular level and eventually use this understanding to improve the efficiency of chemical modification techniques. We explore the relationship between surface modification and the organisation of the adsorbed surfactant for the first time, by combining neutron reflectivity (NR) and atomic force microscopy (AFM), to resolve the precise vertical and surface lateral distribution of the surfactant in or on the polymer. We further consider the resilience of the surface modification and the underlying surfactant structure by studying the same films with these techniques after heating and after rinsing, which are necessary steps towards understanding how to achieve surface modification efficiently. 

## 2. Materials and Methods

### 2.1. Materials and Sample Preparation

*Cis-*Polyisoprene *T*_m_ ~ 24 °C, *T*_g_ = −65 °C, (Supplementary Information Figures S1 and S2, respectively) (Sigma-Aldrich, Gillingham, UK, 97% *cis*-1,4) and C_12_E_5_ (Sigma Aldrich, Gillingham, UK) were purchased and used as received. Deuterated C_12_E_5_ (d_25_-C_12_E_5_) was synthesised at the Rutherford Appleton Laboratories as described elsewhere [25]. Films were spin-cast onto clean silicon wafers, which were first washed with acetone to remove hydrophobic impurities. *Cis*-PI was dissolved in toluene by heating to 50 °C with stirring to yield 4% (w/w) solutions. C_12_E_5_ and d_25_-C_12_E_5_ were also dissolved in toluene to make 10% (w/v) solutions. These solutions were then mixed and further diluted with toluene to yield 2% (w/w) solutions with the desired proportions of polymer and surfactant. Deuterium-labelled d_25_-C_12_E_5_ films were used for neutron and ion beam experiments and hydrogenous samples used for all other experiments. The mixed solutions were heated to 60 °C and spin-cast onto silicon wafers that had been pre-heated to approximately 80 °C. Heating was necessary to avoid the solution de-wetting the substrate during spin-coating. The evaporation phase of spin coating was completed within 30 s. Films were prepared in the same way for NR except silicon blocks with 55 mm diameter and 5 mm thickness were used as substrates instead.

### 2.2. Neutron Reflectometry

Neutron reflectometry (NR) has been used extensively [26] to study polymer films [27] and surfactants in solution [25]. It has also been used successfully to study adsorption of surfactants [28] and migration of oligomers [29] at the surface of thin films, which are particularly relevant to the work that we report on here. This technique is advantageous when studying soft matter as it is possible to conduct experiments at atmospheric pressure and has a vertical resolution (∼0.5 nm, limited by the beam collimation and the size of the silicon blocks used) capable of resolving structure changes on the molecular scale. Concentration versus depth profiles were obtained using the INTER reflectometer at the Rutherford Appleton Laboratories, Chilton, UK. Deuterium labelling provided contrast between layers within the sample by utilising the difference in scattering length density (SLD) of the components. The SLDs of each component of the films and substrates are given in Table 1. A momentum transfer (*Q*) range of 0.005 < *Q*/Å^−1^ < 0.3 was collected to give a complete reflectivity curve. This range requires measurements at 3 angles and has a significant collection time (∼1.5 h per sample). Samples were heated using a temperature-controlled stage and were rinsed under flow of ultrapure water (resistivity: 18.2 MΩ cm) for 10 s. For some samples the rinsing process was repeated for an additional 10 s and the reflectivity measured after a total of 20 s rinsing. Analysis of all reflectivity was performed using MUSCtR [30]. This software allows for the gradual variation in film thickness present in some films, by fitting of multiple depth profiles (Figure S3, Supplementary Material Information Material) to a single set of data. These depth profiles are identical except for a defined variation in thickness of the bulk layer thickness, ± 250 Å, to approximate the macro-scale film thickness undulations. Combining calculated reflectivities in this way suppresses the Kiessig fringes, whilst maintaining the overall shape of R(Q), which cannot be achieved by increasing the interlayer roughness for a single composition profile.

### 2.3. Nuclear Reaction Analysis

Nuclear reaction analysis (NRA) is a real-space depth profiling technique, which can yield similar composition versus depth profiles to neutron reflectometry. NRA was used to assist the interpretation of NR data by providing unambiguous composition profiles, albeit at lower resolution. Films were prepared in the same way as for NR, except that they were spin-cast onto silicon wafers. A 2 mm diameter 0.7 MeV beam of ^3^He^+^ ions was used at a grazing incidence of 70° to the sample normal, to induce nuclear reactions with the deuterons of the d_25_-C_12_E_5_. Data were summed from measurements over several 6 × 2 mm elliptical patches. Backscattered protons were detected at 170° to the incident beam. The NRA technique and instrument setup used are discussed in greater detail elsewhere [31]. Data were fitted using Datafurnace Software [32,33]. Typical data, fits and derived concentration profiles are given in supplementary information, Figure S4.

### 2.4. Atomic Force Microscopy

The surfaces of spin-cast films were analysed using a Bruker Multimode 8 scanning probe microscope in PeakForce quantitative nanomechanical mapping mode (QNM) to map variations in height and mechanical properties simultaneously. Using a J-type scanner, 30 µm × 30 µm areas of each sample were scanned, capturing images with 512 points per line. NuNano Scout 70R probes with a spring constant of 2 N m^−1^ and resonant frequency of 70 kHz were used. Height map data were then flattened using Gwyddion (v2.53) to remove natural curvature caused as the sample is moved relative to the cantilever. AFM was also used to characterise total film thickness by measuring the height difference between the film surface and the substrate where the film was removed by scratching. 

### 2.5. Water Contact Angle Analysis

Contact angle measurements were made using the sessile drop technique with a contact angle goniometer. Images were recorded using a digital camera equipped with a telecentric lens to remove the effects of the depth of field. A 5 µL droplet of the probe liquid (ultrapure water) was placed on the spin-cast film surface and a video of the droplet was recorded. Frames were extracted from the video at regular time intervals and analysed using the DropSnake plugin within ImageJ [34]. 

## 3. Results

### 3.1. Additive Segregation

**Figure 1:**
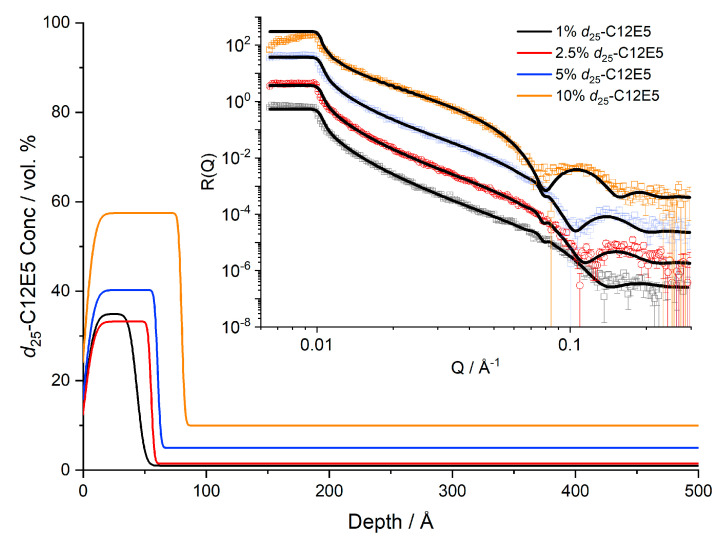
Near-surface concentration profiles of d_25_-C_12_E_5_ in *cis*-PI at 20 °C. Reflectivity data and fits are shown in the inset, offset for clarity.

NR results are presented as volume fraction depth profiles (Figure 1). The surface excess provides a convenient measure of the total amount of segregated surfactant at the exposed surface. Here, we define the surface excess, z^*^, which is equivalent to the thickness of a pure layer of surfactant, as

(1) $$z^{*}=\int_{0}^{\infty }\varphi \left(z\right)-\varphi_{b}dz$$

where φ(z) is the concentration at depth z and $\varphi_{b}$ is the bulk volume fraction. This measure of the surface excess is related to the area per adsorbed molecule, more commonly used in the surfactant science literature, *Γ*, by

(2) $$\Gamma =z^{*}/v_{surf}$$

where $v_{surf}$ is the volume of the surfactant, ~701 Å^3^.

**Figure 2:**
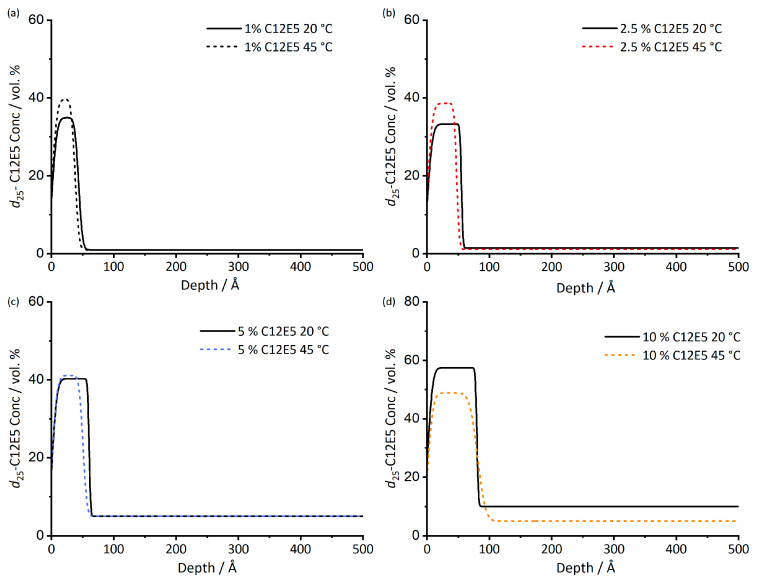
Depth profile of d_25_-C_12_E_5_ in *cis*-PI films at both 20 and 45 °C. The depth profiles are determined from the scattering length density profiles of the films. Surfactant loadings are 1% (**a**), 2.5% (**b**), 5% (**c**), 10% (**d**). Fitted data used to obtain 45 °C depth profiles are given in Figure S5.

Figure 1 and Figure 2 present the concentration dependence of the surfactant in the near-surface region of the film. The total film thickness measured by AFM was 4500–6000 Å; therefore, too thick to characterise directly by NR. We found clear evidence of d_25_-C_12_E_5_ blooming to the surface of PI films both below and above the melt temperature of PI. In all cases the films are well above their *T*g of ~−65 °C, Figure S3. Whilst the bulk shows a concentration consistent with the expected average surfactant loading, there is a much larger concentration present near the surface. As the average concentration increases, the surface layer also becomes thicker and more enriched. However, the maximum surface concentration of the surfactant is significantly less than 100% and the surfactant instead presents as an enriched layer in the depth profile. This enriched surface layer is also confirmed in nuclear reaction analysis depth profiles, where a layer of d_25_-C_12_E_5_ is also present even at lower resolution (Figure S4). Computational studies have calculated the length of a C_12_E_5_ molecule to be approximately 28 Å in a vacuum [35]. This molecular length represents the longest possible length of the molecule: whilst the length can be decreased by folding or tilting of the molecule, it cannot exceed this length. It is clear that the adsorbed surface layers are much thicker than the length of a single molecule, Figure 1. Furthermore, samples of 10% surfactant loading show a surface excess of 36 Å (Table 2), which exceeds the maximum quantity of surfactant that could exist as a single layer. Our results clearly show that while it is commonly the case that there is sufficient bloomed surfactant on the film surfaces to form a pure monolayer, it takes the form of incomplete layers with a surface concentration of 60% surfactant or less. 

### 3.2. The Effect of Temperature Elevation on the Surface Excess

Upon heating, the surface excess shows very little change, Table 2. The uncertainty of surface excess measurements is 1–3 Å, [36] thus, there is no evidence for the surface excess measurements given in having any significant temperature dependence. The concentration profiles from which z* values were derived are shown in Figure 2. Although there appears to be some small variations in the calculated profiles, these too are close to the limit of precision of the measurement, and there is no systematic shift in the surface concentration or thickness of the adsorbed layer with changing temperature. We note that in this temperature range, none of the film components are solid or glassy; therefore, samples should be relatively unhindered in approaching equilibrium.

### 3.3. Water Exposure Effects on the Surface Excess

The surface segregation of d_25_-C_12_E_5_ was quantified using NR at 45 °C and 60 °C and then after repeated 10 s rinses with ultrapure water and these surface excess measurements are summarised in Table 2. Rinsing once or even twice causes surprisingly little change in surface excess: the largest change shown upon rinsing is in the 5% surfactant sample, with a decrease of ~27% (between 60 °C and re-measured at 20 °C after a 10 s rinse). When compared to the uncertainty of these measurements, noting the slightly higher values recorded after a second rinse, there is again remarkably little change in surface excess concentration upon rinsing. 

Wahlgren et al. [37] determined the solubility of C_12_E_5_ to be in excess of 10 w/v% (based on cloud point). From this value, for a single rinse of approximately 10 mL of water, 1 g of C_12_E_5_ could be dissolved. Based on the surface excess thickness for 1% C_12_E_5_, loading a 55 mm diameter silicon block will have 3.2 µg of surfactant at the surface. This would only require 32 nL of water to dissolve, meaning that there is over 300,000 times the amount of water required to remove all of the surface layer at equilibrium present. If all of the surfactant was capable of moving from the film into the water, it would be reasonable to assume that all surfactants would be removed from the surface: the surface layer is thin and so there is little to prevent surfactant loss. However, the post-rinsing NR results for the surface excess, summarized in Table 2 indicate that the surfactant/polymer film is remarkably stable with respect to rinsing. Further rinsing for an additional 10 s appears to have no significant effect. 

### 3.4. Water Contact Angle Analysis

Our water contact angle (WCA) results show that significant levels of hydrophilicity are generated in films containing at least 5% C_12_E_5_ surfactant. It is interesting to note that while the surface excess measured by NR at 5% surfactant is similar to results for lower loadings of surfactant, there is a significant change in WCA over this range. 

**Figure 3:**
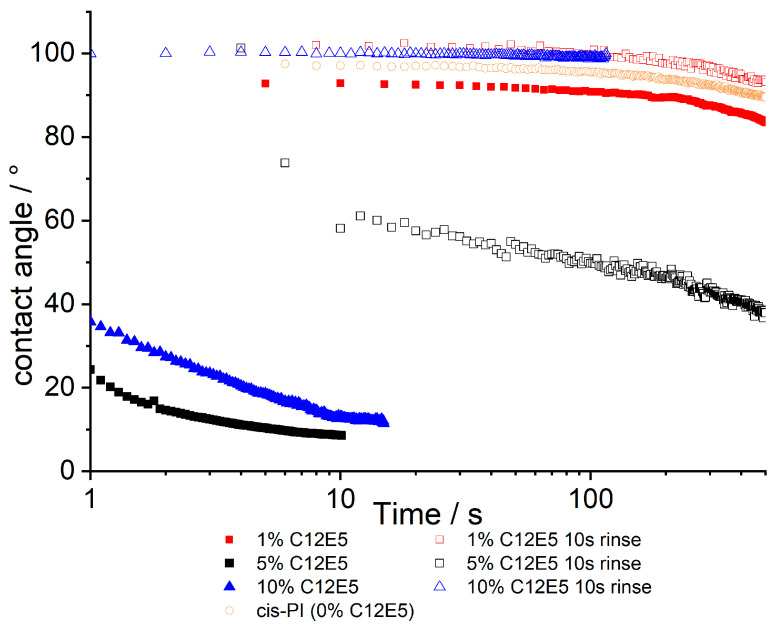
Contact angles for *cis*-PI films containing between 1 and 10% C_12_E_5_ both before and after a 10 s rinse with deionised water. The data are a function of time where 0 is the time that the probe droplet (water) is placed on to the surface. A *cis*-PI sample has also been shown in orange, showing 0% surfactant loading.

Whilst the NR data shows that the adsorbed surfactant is stable with respect to water exposure, the contact angle data presented in Figure 3 presents equally clear evidence that the surface properties are dramatically modified by rinsing. It should be noted that contact angle measurements show significant (several degrees, Figure S9) variation, when repeat measurements are carried out, even when on nominally identical samples. Thus, we treat individual profiles with caution, while noting the following points: In the as-made samples, there is no significant surface modification at 1% C_12_E_5_ compared to the 0% control sample. Contact angles are relatively independent of time, only decreasing after periods of >100s, at which point evaporation of the contacting droplet gives rise to a receding contact angle, which is lower than the initial advancing contact angle. At higher (5%, 10% C_12_E_5_) loadings, film surfaces are much more hydrophilic and contact angles decrease with time over short periods. It is expected that the hydrophilicity of the films will depend on the presence of C_12_E_5_ on the surface of *cis*-PI, which is otherwise hydrophobic. After rinsing, the contact angles increase for all compositions, and in some cases (1 and 10% C_12_E_5_) show contact angles greater than 90°. When comparing the results from NR and contact angle analysis, there is evidence for incomplete surface coverage: NR shows there is a layer enriched in surfactant for all samples, yet not all samples show wetting behaviour when water is placed on the surface. This is indicative of domains of surfactant on the surface with other regions of exposed *cis*-PI. 

### 3.5. Surface Structure Change after Water Exposure

**Figure 4:**
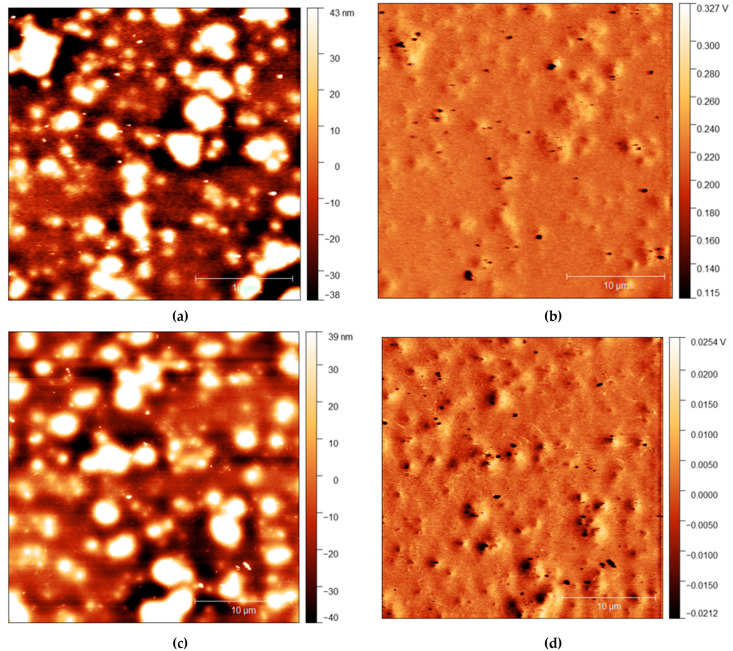
(**a**) AFM height map of *cis*-PI films with 0% loading of C_12_E_5_ and (**b**) Adhesion maps for the same position on the same film; (**c**) height map of the same film shown in the top AFM images after 10 s rinse and (**d**) the adhesion map.

**Figure 5:**
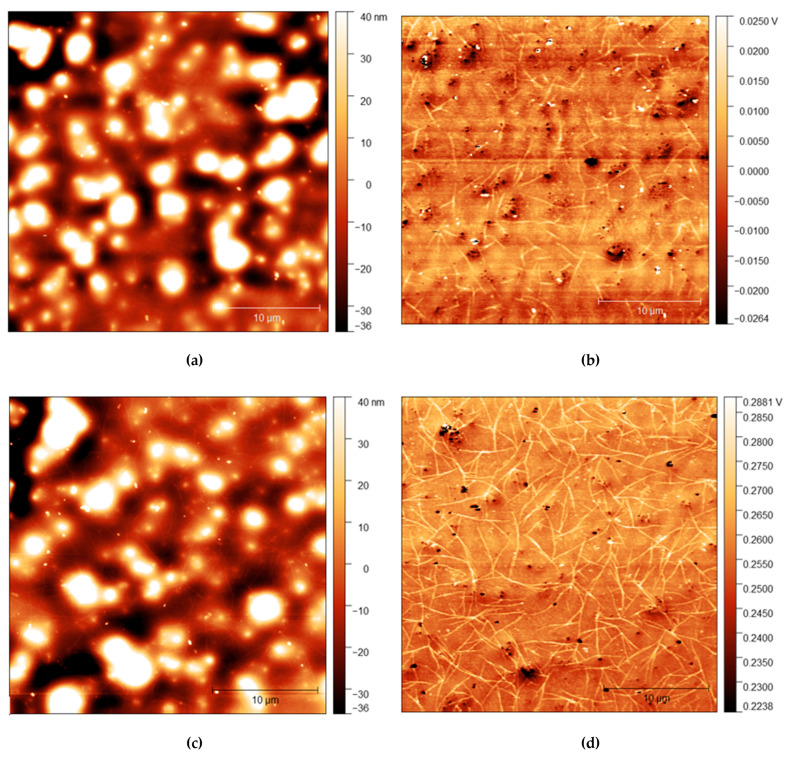
(**a**) AFM height map of *cis*-PI films with 2.5% loading of C_12_E_5_ and (**b**) Adhesion maps for the same position on the same film; (**c**) height map of the same film shown in the top AFM images after 10 s rinse and (**d**) the adhesion map.

**Figure 6:**
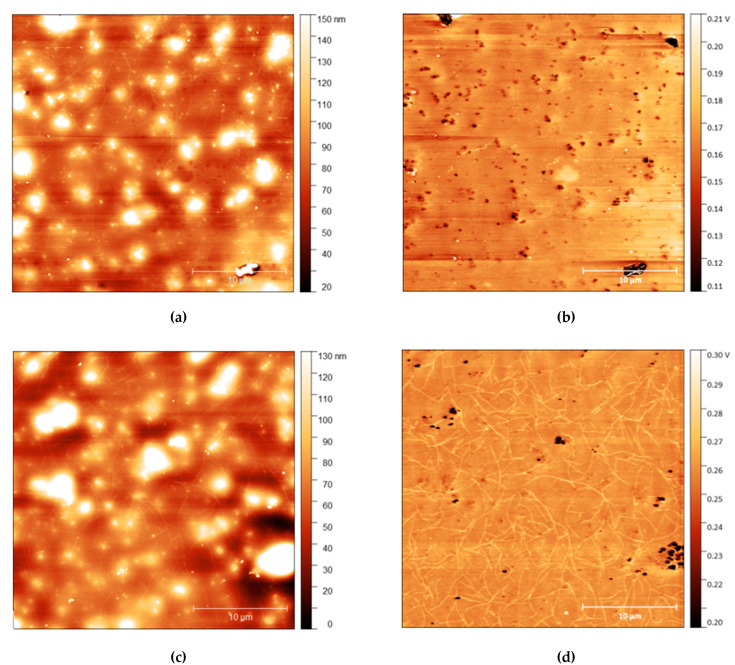
(**a**) AFM height map of *cis*-PI films with 10% loading of C_12_E_5_ and (**b**) Adhesion maps for the same position on the same film; (**c**) height map of the same film shown in the top AFM images after 10 s rinse and (**d**) the adhesion map.

AFM measurements are shown in Figure 4, Figure 5 and Figure 6 with height and adhesion maps presented. The AFM images confirm the presence of gradual undulations in film thickness, which necessitated multi-profile fitting of the NR data. Before rinsing, a small number of thread-like “strand” features were found on the surface. These strands are slightly raised with respect to the surface and correlate with regions of increased adhesion. In the absence of any variation in composition, raised features sometimes correlate with a reduction in adhesion due to the reduced contact area between the probe and the surface. Here, the increase in adhesion on these raised features indicates that the strands are of a different composition from the surrounding surface, most likely the surfactant.

The strands become more apparent at higher concentrations, with strands appearing in the adhesion surface maps above 2.5% before rinsing. The strands also become more pronounced after rinsing for 10 s, with more strands appearing on the surface after rinsing. It is important to note, however, that images do not show the same position on each sample. As the sample is removed from the AFM to be rinsed, it is not possible to return the sample to the same position upon reintroducing it to the microscope. However, the trends observed are consistent across all samples. 

## 4. Discussion

### 4.1. Surfactant Blooming

There are few literature examples of hydrophilic additives being used to within non-polar polymers. Whilst Zhu and Hirt reported the formation of a surface layer of hydrophilic additives present on a hydrophobic polypropylene film [19], this work could not resolve the precise thickness of this surface layer because of the more limited depth resolution of ATR-FTIR used. Nevertheless, it appears that hydrophilic modification is possible, not only in the molten extruded films of Zhu and Hirt’s study, but also in spin cast films that we consider here. This similarity suggests that the preparation technique and particularly the presence of solvent in sample preparation does not hinder the migration of an additive molecule.

Studies by Briddick et al. [31] in polyvinyl alcohol films showed surface layers of C_12_E_5_ present with a thickness that is comparable to the thicknesses reported in Figure 1, suggesting that the initial surfactant segregation is similar in both PVA and *cis-*PI matrices. The surface segregation in *cis*-PI is much less expected than in PVA: the PVA films were spin-cast from water in which these surfactants are highly surface-active, but these PI films are spin-cast from toluene, with a low surface energy and so no surface activity of C_12_E_5_ is expected. Furthermore, the surface energy of PI (32 mN m^−1^) [38] is lower than that of PVA (37–59 mN m^−1^) [39,40,41], again indicating a lower thermodynamic impetus for surface segregation.

The thickness of the enriched surface layer is well-characterised by NR and presents evidence of a multilayer structure. Multiple (even number) layers of surfactant would allow the surfactant to present the hydrophobic, low surface energy groups to both the hydrophobic polymer and the air. Whilst an even number of layers could present a low energy surface to both the air and bulk polymer, when exposed to water, only an odd number of layers would maintain the arrangement at the cis-PI whilst presenting a hydrophilic surface to the water interface. It is interesting to note that the smallest layer thickness observed is ~40–50 Å, corresponding to a bilayer structure, possibly tilted. Furthermore, the 10% sample shows a surface layer that is nearly 100 Å thick, corresponding closely to a 4-layer structure. No samples show a surface layer with a thickness between these values, suggesting that a 3-layer structure is unstable. Such a structure would have to present polar oxyethylene surfaces against the non-polar *cis*-PI or air, neither of which would be energetically favourable. The small discrepancy between the measured layer thicknesses and even multiple of the extended surfactant chain length indicates some tilting of the surfactant molecule orientation with respect to the sample normal, or possibly some overlap or interdigitation between layers.

Although the temperature dependence of surfactant segregation in polymers has received relatively little attention, we can draw some useful comparison between our experiments and previous reports on the behaviour of plasticisers in polymers. Xie et al. [42] investigated surface segregation of dioctyl phthalate (DOP) to the surface of polystyrene (PS) films. They found that heating samples after film preparation caused an increase in the surface DOP concentration up to 60 °C, after which surface DOP concentrations decreased. The samples were below the *T*_g_ of PS in this example, and heating towards the *T*_g_ likely favours the equilibration of the DOP, promoting segregation. However, because the *cis*-PI samples are far above their *T*_g_, heating has little effect on the vertical distribution. The absence of any strong temperature-dependence in these films suggests that heating has little impact on the thermodynamic favourability of adsorption over this range.

The concentration-independence of z^*^ is unexpected for an incompatible surfactant that forms multilayers on the film surface. Previously, we have found that highly compatible amineoxide surfactants in PVA form a concentration-independent surface excess, but the segregation of other, less compatible surfactants is strongly dependent on concentration. [36]. In the case of compatible mixtures, z^*^ and the thickness of the adsorbed layer corresponded closely to a surfactant monolayer [36]. Here, however, the multilayer adsorbate is not consistent with a compatible surfactant and as such, we would expect the amount of surfactant excluded to the surface to increase with increasing bulk concentration.

The fraction of surfactant within the entire film that appears as an enriched layer is also a useful metric to assess. This fraction can be defined by Equation (3), where $\varphi_{\mathrm{surf}}$ is the surface fraction, $z^{*}$ is the surface excess and $\varphi_{\mathrm{total}}$ is the total amount of surfactant within the film of thickness, *L*.

(3) $$\varphi_{\mathrm{surf}}=\frac{z^{*}}{L\varphi_{\mathrm{total}}}$$

Throughout the composition range studied, the majority of the surfactant remains in the bulk of the film, Table 3. This new observation indicates that the surface modification is not very efficient at a molecular level for these materials and concentration range, but it does allow for the possibility that the remaining surfactant in the bulk could later be exploited to regenerate a hydrophilic surface. Such a process might be possible if further surface segregation could by triggered by some stimulus, such as heating.

When considering the effect of temperature elevation, it is important to consider the effect of evaporation of the surfactant on the distribution. This is because the combination of a minute sample volume (~0.1 mg) with a macroscopic surface area (~10 cm^2^) in these spin-cast films provides an ideal geometry to promote evaporation. In a similar thin-film geometry, Smith et al. [43] showed that for a small molecule (a plasticiser) in a polyester/polyurethane film, the rate of loss of the surfactant is limited not by the rate of diffusion of the molecule in the film, but by the rate of evaporation of additive from the film. We note for our purposes that if the rate of evaporation were significant compared to the rate of diffusion within the film, then the near-surface region of the film would be depleted, rather than enriched in surfactant as we found. Furthermore, we find that even after equilibrating samples at 45 °C for approximately 8 h, the changes in the composition profile are negligible; therefore there is no evidence for evaporation during heating.

### 4.2. Surface Structures

When the hydrophobic surfaces observed in contact angle measurements are compared with the surface excesses before and after rinsing, there is evidence for molecular reorganisation following initial exposure to water to render the surface more hydrophilic, but after this exposure, the surface then becomes quite hydrophobic with respect to further contact with water. This suggests some further rearrangement after drying, from which the surfactant is then unable to reorganise into a hydrophilic coating when the contact angle experiment is repeated after rinsing. C_12_E_5_ has previously been shown to form lamellar structures at high concentrations in water [44]. Whilst the substrate on which these structures form is significantly different, with a liquid interface compared to a polymer, it is possible that a similar structure forms at the surface of the polymer, minimising contact of hydrophilic head groups with a low surface energy polymer, and simultaneously presenting a low surface energy interface to the air.

The precise origin of the surface changes as seen in Figure 4, Figure 5 and Figure 6 is unclear, but is consistent with some change in the organisation of the surfactant. While strand-like features of C_12_E_5_ adsorbed on mica surfaces under water have been reported by Dong and Mao [45], those features, which are quite closely packed and aligned parallel to each other are very different from the more freestanding extended features that we have observed here. We are unaware of any similar behaviour reported as spontaneously forming at the solid-air interface. In our work, the strands that spontaneously form are much longer than have previously been reported on surfaces and are unusual that they are essentially free-standing and do not exist in arrays and are not obviously altered by rinsing. We note that although these surface features are much straighter, and possibly branched, the length of the strand features is roughly consistent with the worm-like micelles of C_12_E_5_ found in solutions by light-scattering studies [46].

Our results show an interesting discrepancy between the surface excess measured by NR, which is remarkably stable with respect to rinsing, and the wettability behaviour, which is altered by rinsing. The fact that repeated rinsing has no significant effect on the surface layer measured by NR suggests that it remains in place rather than is removed, then replaced from the bulk. Had the latter process occurred, we would expect that with two rinses, there would be a systematic depletion in any regenerated surface layer. Overall, our results indicate that the resilient surface excess measured by NR is not entirely responsible for the observed wetting behaviour. Interestingly, the surface excess measured by nuclear reaction analysis, 69 Å, is almost twice the value measured by NR. We postulate that this discrepancy arises because the film surface includes some C_12_E_5_ that is detected by NRA, but not NR, and it is this fraction of the segregated surfactant that is mainly responsible for the wettability phenomena. The contribution to the surface excess that is only detected by NRA is most likely to arise from the ‘strands’ that are detected by AFM, and may also account for the anomalous concentration dependence of the surface excess noted earlier. These strands are relatively small (several microns) and are likely to be smaller than the coherence length of the neutrons, meaning that very little of the NR profile observed arises from incoherent addition.

## 5. Conclusions

We have shown that for spin-cast C_12_E_5_/*cis*-PI blends, a rich variety of complex and intriguing blooming behaviour is found. A clear surface excess of the amphiphile presents at the air interface, which is nearly invariant over a significant range of composition and temperature. There are few precedents for amphiphile segregation at hydrocarbon polymer surfaces [22,23,24] and neither concentration or temperature dependence of adsorbed nanostructures nor their stability with respect to rinsing have previously been reported. Our work shows unexpected concentration dependence and resilience with respect to rinsing, which is important for applications based on this model system. Even when these blends are spin-cast from a low surface energy solvent, non-ionic surfactant adsorption is spontaneous, showing that this behaviour is more general than was previously known. We have also demonstrated that the non-ionic surfactant, which adsorbs to the surface is resistant to evaporation, with a surface excess remaining after temperature elevation for several hours.

The NR depth profiles presented indicate that surface adsorption is persistent even after exposure to significantly more water than is required to remove the surfactant. However, the contact angle measurements of the films before and after rinsing indicate a change in the surface properties of the film, indicating that there must be a structure change in the surfactant that occurs upon exposure to water. We can resolve this difference in observations by recognising that NR is only sensitive to planar structures such as complete surface layer. Other surface features such as strings and droplets are not detected by specular NR, meaning that if these structure changes are induced upon water exposure, we may see little evidence of these features in NR, even though NR provides a very precise characterisation of surfactant bloomed on polymer surfaces. Only by a combination of techniques can an accurate picture of the blooming process be obtained, and it is apparent that this level of detail is needed in order to understand why such a complex relationship between surface segregation and surface modification exists.

## Figures and Tables

**Table 1:** Scattering Length Densities for Hydrogenous and Deuterated Components.

Component	SLD/10^−6^ Å^−2^	Density/g cm^−3^
*cis*-PI	0.26	0.91
d_25_-C_12_E_5_	3.60	0.963
Si	2.07	2.33
SiO_2_	3.47	2.56

**Table 2:** Surface excess for samples at 20, 45 and 60 °C and after rinsing for 10 s and 20 s with deionised water. NR data and fits used to obtain depth profiles are presented in Figures S5–S8.

	z*/Å				
[d_25_-C_12_E_5_] /wt%	20 °C	45 °C	60 °C	20 °C, 10 s rinse	20 °C, 20 s rinse
1	14	13	14	13	14
2.5	16	17	14	11	11
5	15	17	18	13	14
10	36	34	33	30	34

**Table 3:** Surface fractions for *cis*-PI/C_12_E_5_ at 20 °C.

Film Surfactant Loading/wt. %	$\varphi_{\mathrm{surf}}$
1	0.24
2.5	0.20
5	0.08
10	0.07

## Data Availability

Data for the neutron reflection experiments is available at https://doi.org/10.5286/ISIS.E.RB1910298.

## Supplementary Materials

The following are available online at https://www.mdpi.com/article/10.3390/polym13193407/s1, Figure S1: DSC Thermogram of cis-PI heated at 10 °C per min. The peak with a maximum at 24.38 °C shows the melting transition of the cis-PI, Figure S2: Raw DSC data of cis-PI, showing the glass transition at −65.08 °C, calculated from the extrapolated half C_p_ point, Figure S3. (a) sld profile for 1% C_12_E_5_ in cis-PI at 20 °C illustrating how the data are fitted. The three sld profiles shown have different bulk layer thickness and the difference in thickness between each profile is 250 Å. (b) The averaged predicted reflectivity profile from each of these model sld profiles, which was fitted to the experimental data, Figure S4. Nuclear reaction analysis depth profile of 10% d_25_-C_12_E_5_ in cis-PI showing a surface excess. Inset: Fitted NRA data used to obtain the depth profile, Figure S5. Two successive measurements of contact angle on distinct locations on a single 1% C_12_E_5_/cis-PI sample. Figure S6. NR data and fits for 1 to 10% d_25_-C_12_E_5_ in cis-PI at 45 °C, Figure S7. NR data and fits for 1 to 10% d_25_-C_12_E_5_ in cis-PI at 60 °C, Figure S8. NR data and fits for 1 to 10% d_25_-C_12_E_5_ in cis-PI after a 10 s rinse, Figure S9. NR data and fits for 1 to 10% d_25_-C_12_E_5_ in cis-PI after a 20 s rinse.
