# Supplementary material for: Water-Resistant Surface Modification of Hydrophobic Polymers with Water-Soluble Surfactant Additives
Source: Polymers (Basel). 2021 Oct 3;13(19):3407. doi: 10.3390/polym13193407 (PMC8512660; doi:10.3390/polym13193407)
Supplement: Supplementary file 1 [file polymers-13-03407-s001.zip › polymers-1402329-supplementary.pdf]

polymers

Supporting Information

Water-Resistant Surface Modification of Hydrophobic Polymers with Water-Soluble Surfactant Additives

Colin P. Gibson [1], Matthew A. Litwinowicz [1], Rebecca J. L. Welbourn [2], Maximilian W. A. Skoda [2], Jan Claussen[3] and Richard L. Thompson [1,*]

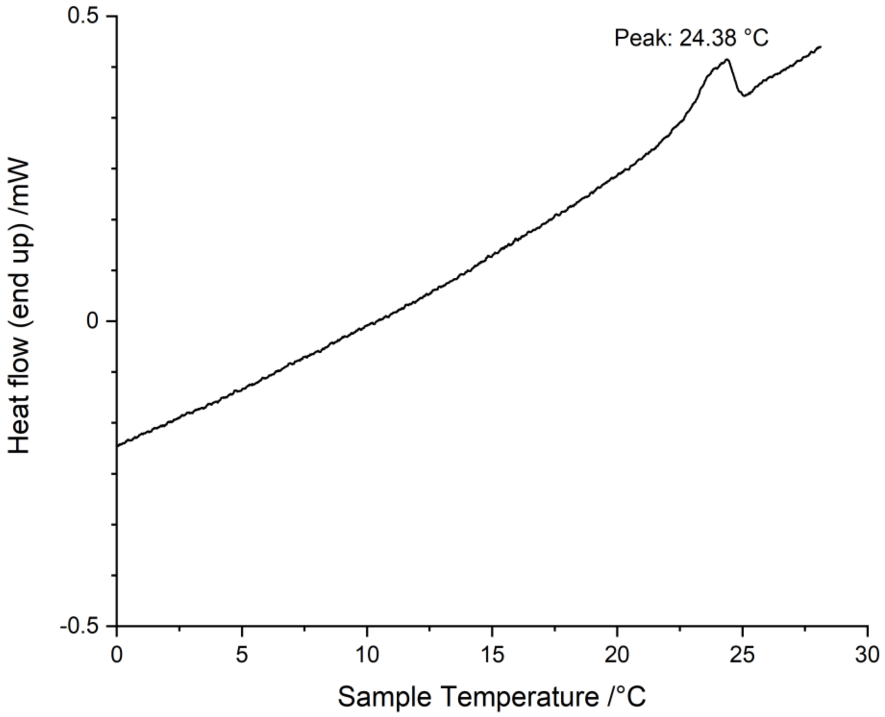

Figure S1. DSC Thermogram of cis-PI heated at 10 °C per min. The peak with a maximum at 24.38 °C shows the melting transition of the cis-PI.

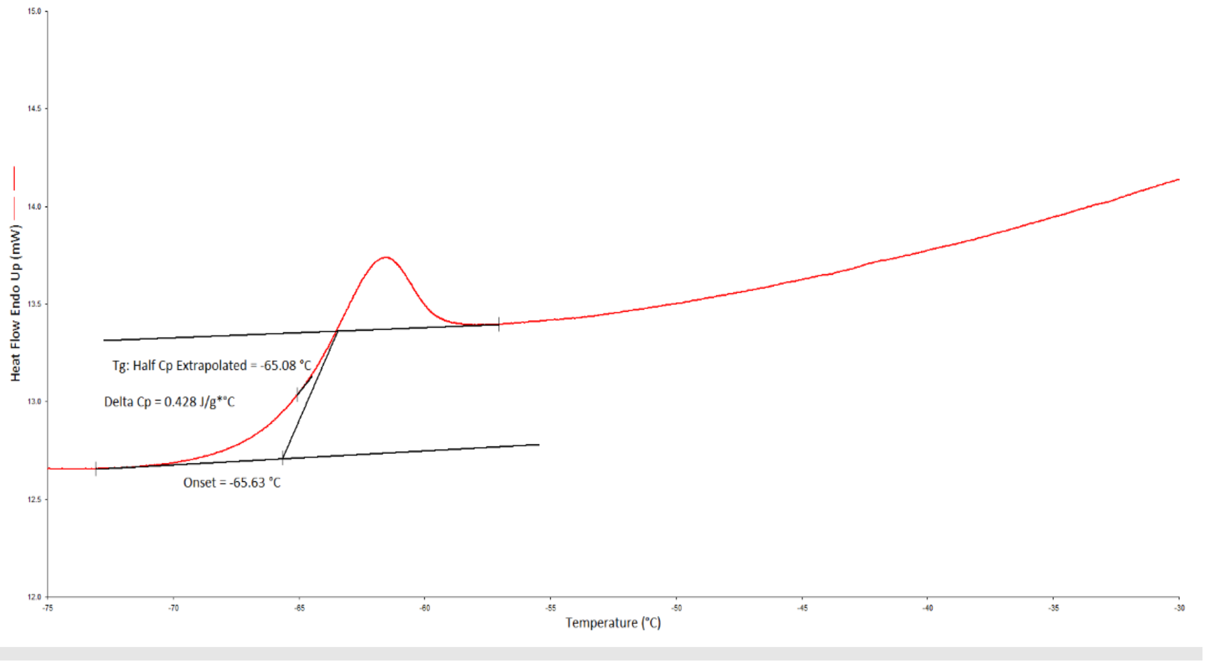

Figure S2. Raw DSC data of cis-PI, showing the glass transition at -65.08 °C, calculated from the extrapolated half C_p point.

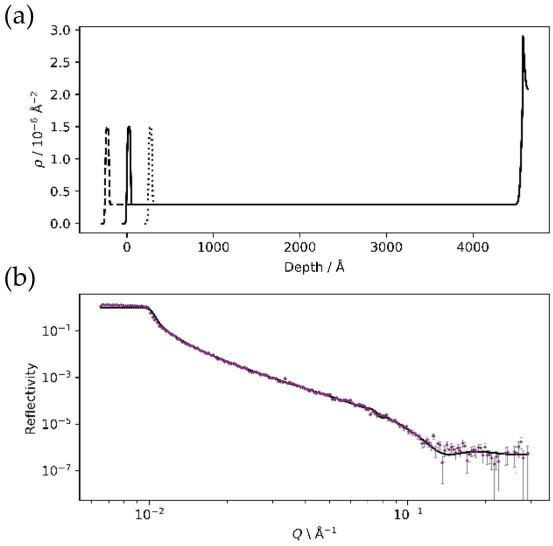

Figure S3.(a) sld profile for 1% C12E5 in cis-PI at 20 °C illustrating how the data is fitted. The three sld profiles shown have different bulk layer thickness and the difference in thickness between each profile is 250 Å. (b) The averaged predicted reflectivity profile from each of these model sld profiles, which was fitted to the experimental data.

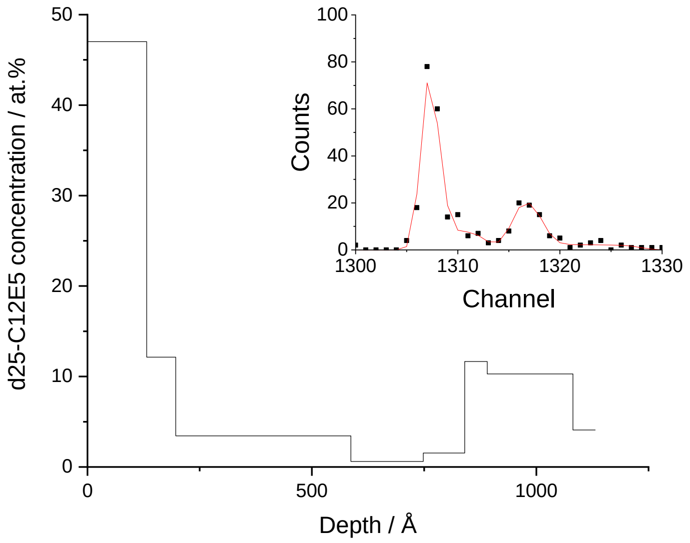

Figure S4. Nuclear reaction analysis depth profile of 10% d_{25}-$C_{12}E_5$ in *cis*-PI showing a surface excess. Inset: Fitted NRA data used to obtain the depth profile.

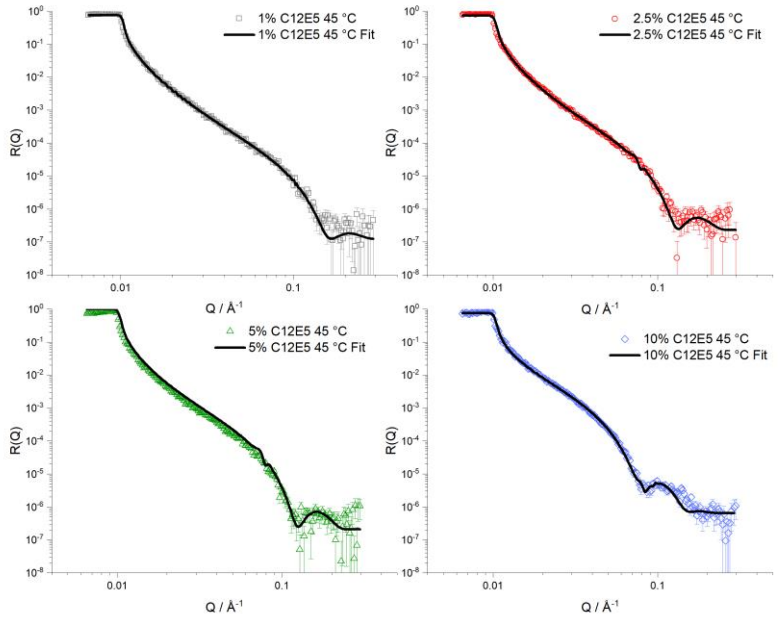

Figure S5. NR data and fits for 1 to 10% d_{25}-$C_{12}E_5$ in *cis*-PI at 45 °C.

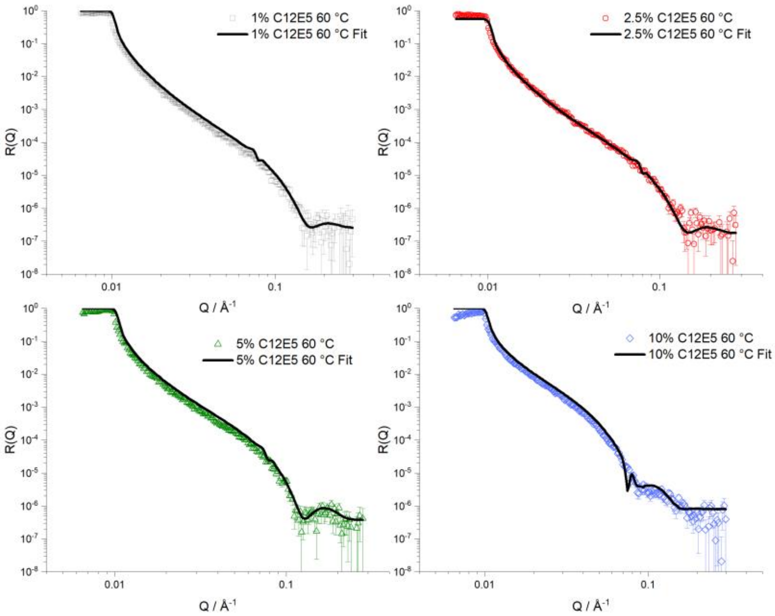

Figure S6. NR data and fits for 1 to 10% d_{25}-$C_{12}E_5$ in *cis*-PI at 60 °C.

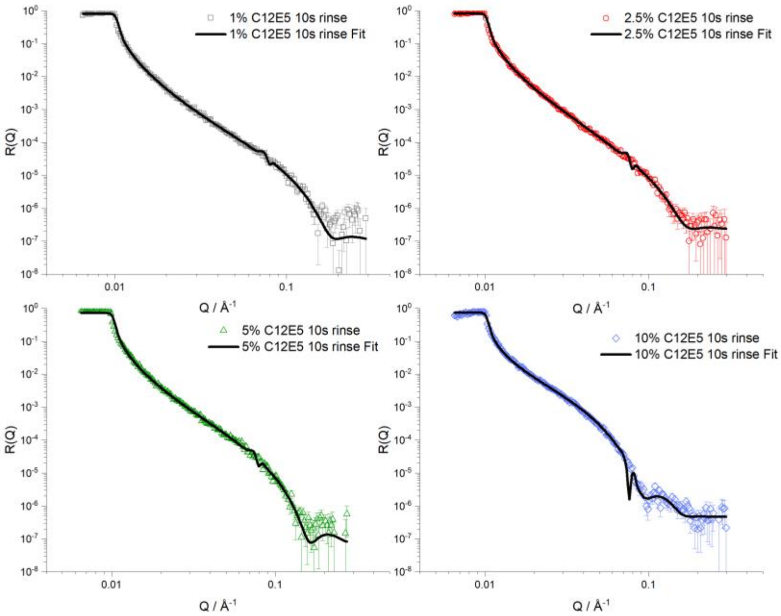

Figure S7. NR data and fits for 1 to 10% d25-C12E5 in cis-PI after a 10s rinse.

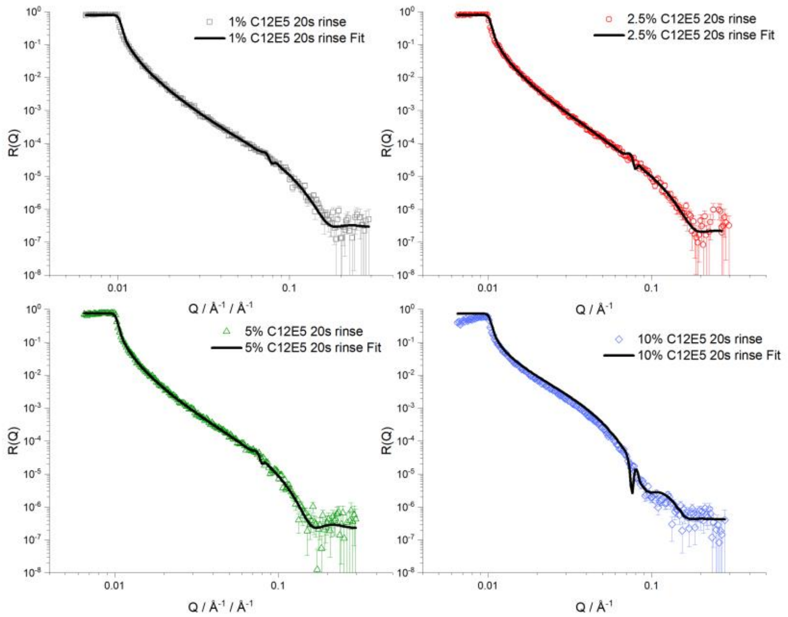

Figure S8. NR data and fits for 1 to 10% d25-C12E5 in cis-PI after a 20s rinse.

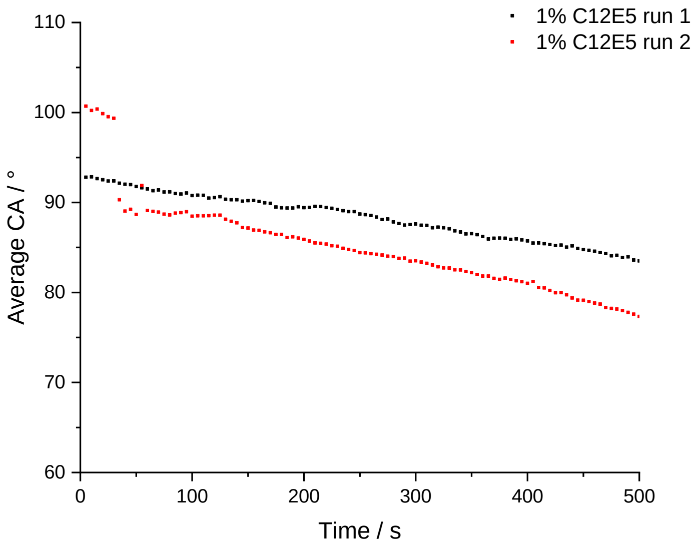

Figure S9. Two successive measurements of contact angle on 2 distinct locations on a single 1% C12E5 / cis-PI sample, showing the variability of results.
